# Alternative perimetric tests for patients with drug-resistant epilepsy

**DOI:** 10.1371/journal.pone.0318025

**Published:** 2025-02-24

**Authors:** Monika Thakur, Abhinay Kumar Gattu, Jagarlapudi M. K. Murthy, PremNandhini Satgunam

**Affiliations:** 1 Brien Holden Institute of Optometry and Vision Science, Hyderabad Eye Research Foundation, L V Prasad Eye Institute, Hyderabad, India; 2 Department of Optometry and Visual Sciences, School of Health and Psychological Sciences, City St George’s, University of London, London, United Kingdom; 3 Department of Neurology, CARE Institute of Neurosciences, Hyderabad, India; 4 Renova Institute of Neurological Sciences, Hyderabad, India; University of Tübingen, GERMANY

## Abstract

**Objective:**

Visual field assessment is an important presurgical test for patients with drug-resistant epilepsy (DRE), particularly with posterior cortex epilepsy. However, the assessment using conventional perimeters like Humphrey Visual Field Analyzer (HFA) may not always be feasible in some patients. This study aims to determine if alternative methods like tangent screen perimetry or Baby Vision Screener (BaViS) can be used for such patients.

**Methods:**

This retrospective study included 17 patients (mean age: 18 ±  8.7, range: 6 to 38 years) with DRE. Visual fields were attempted first with HFA and then with one or both alternative methods, by different examiners. Visual field extent was measured using the kinetic perimetry mode in the alternative methods. With HFA, kinetic and/or static perimetry was attempted.

**Results:**

Only 12% of the patients were able to perform the HFA. Whereas the testability of BaViS was 91% and tangent screen perimetry was 87%. Comparable visual field isopters were obtained on one patient on whom all the 3 tests could be performed, and in two patients on whom at least two tests could be performed reliably. For one patient, visual field isopters could not be quantified on any device. In this patient, a gross visual field assessment was possible using BaViS.

**Conclusion:**

BaViS or tangent screen perimeter can be used to quantify visual field defects in patients with DRE when conventional perimetry is not possible. Such an approach may help the clinician in assessing the suitability of patients with DRE and visual field deficits, for epilepsy surgery.

## 1. Introduction

Despite the development of several new anti-seizure medications (ASMs) with different mechanisms of action, still about 30% of people with epilepsy may have drug-resistant epilepsy (DRE) [1–3]. Some of these patients may be candidates for epilepsy surgery. Presurgical workup involves several investigations that include assessing the patient for any visual field deficits, particularly in patients with posterior cortex epilepsy (PCE) [4,5]. In a significant proportion of patients with PCE, visual field defects can be present and it could also involve the epileptogenic zone in the brain [6–8]. The types of visual field defects in these patients include quadrantanopia or irregular shaped visual field defects. Postoperatively some patients may develop new visual field defects, particularly quadrantanopia following anterior temporal lobectomy or there may be an increase in the preexisting visual field defects [9,10].

Visual field defects are detected by performing perimetry. It could be challenging to do perimetry, especially in patients with epilepsy who have a short attention span, and a cognitive impairment, who are unable to process instructions, or who simply may not be able to hold their head straight or press a button to give a response. Even the common and simple visual field test used in the clinic, confrontation perimetry, can also be challenging for these patients. Such challenges exist for testing visual fields in infants as well. To overcome many of these challenges, we at LV Prasad Eye Institute, Hyderabad, developed a Pediatric Perimeter (now called the BaViS-Baby Vision Screener), a novel device to measure visual fields in infants and patients with special needs [11–13]. The device uses reflexive eye movements made by the infants towards the presented light stimuli in a dark room, as a surrogate measure for peripheral detection or visual field extent. Therefore, the need for attentively holding a steady gaze is circumvented, since the infants will reflexively fixate only on the light stimuli. The testing position with this device can be supine, and with the silhouette cut in the mattress on which the patient lies down, the pitch, yaw, and roll head movements are minimized. With no requirement either for a verbal response or a button press, and with a relatively stable head position, an estimate of the visual field is still possible for infants. Given that even some pediatric and adult patients with epilepsy may have the same constraints as infants, the BaViS device can be used for them as well.

This study aimed to check the feasibility of testing visual fields in patients with DRE, for whom conventional standard perimetry testing was challenging. In such patients, visual fields were tested using the alternative methods (BaViS or tangent screen perimetry [14]).

## 2. Materials and methods

### 2.1. Patients

This retrospective study included 17 patients with drug-resistant, posterior cortex epilepsy (DR-PCE). The patients were referred by a neurosurgeon to the Low Vision and Rehabilitation Clinic, LV Prasad Eye Institute (LVPEI), Hyderabad. These referrals were made for visual field evaluation, as a part of pre-surgical workup. These patients could not do the conventional perimetric test in their testing site and hence were referred to our institute. The referral period was between 2015 and 2023. Informed written consent was obtained from the parents or caretakers, as even the adult patients were unable to comprehend the form. Consent was also taken for photograph and video recording with the BaViS device (experimental device) if it was used for testing. The institutional review board of LVPEI approved the study protocol (LEC 04-14-045) for testing with BaViS. The study protocol adhered to the tenets of Declaration of Helsinki. The records were reviewed in June 2023 by the first author and the data was recorded anonymously.

### 2.2. Ophthalmic examination

As a part of the regular ophthalmic examination, patients underwent visual acuity assessment using the COMPlog visual acuity electronic chart, non-cycloplegic refraction using retinoscopy, detailed anterior segment evaluation using slit-lamp biomicroscope, and intraocular pressure assessment using Goldmann Applanation tonometry. Eye alignment was assessed using the Hirschberg corneal reflex test, and extraocular motility was evaluated using the Broad-H test. The visual field was assessed both with standard (when possible) and alternative techniques of perimetry. All the perimetry techniques are explained below.

### 2.3. Visual Field examination

#### 2.3.1. Humphrey visual field analyzer (HFA).

For those patients who could follow instructions, HFA was attempted first. Patients were instructed to keep their head stable on the chin rest and to maintain steady fixation on the central target. Patients were asked to press the button only when they detect a light stimulus in the periphery and not to move their eyes toward the target. Different testing algorithms (30-2, 60-4, 120-2, Esterman field test, and kinetic mode) are available in HFA. To assess in natural viewing condition, the Esterman visual field test can be considered, as it presents suprathreshold targets in binocular viewing condition [15]. The kinetic mode (i.e., targets move from periphery to center) also allows some flexibility for the examiner to manually choose and present the targets. If the test result showed an advanced visual field defect or the test was unreliable, or if the patient was unable to perform the test, then other behavioral perimetry techniques (tangent screen perimetry or the Baby Vision Screener) were further attempted to investigate the visual field extent.

#### 2.3.2. Tangent screen perimetry.

Tangent screen perimetry is a manual kinetic perimetric procedure. For the purpose of our testing, the patient was seated at 50 cm in front of the tangent screen, to allow the peripheral visual field of about 43° extent to be measured, as opposed to the standard 1-meter testing distance that permits only 25° to be measured. A 3 mm white spot on a black wand was moved from the non-seeing periphery towards the center by an experienced optometrist, along the radial lines marked on the tangent screen. The patient was instructed to maintain an upright posture with their head facing towards the screen and to fixate on the central fixation target on the screen. The patient was instructed to say “yes” when the spot on the wand was visible. The position on the screen where the spot was visible was marked on a recording sheet. All the marked points were connected to plot the visual field isopters of the patient [14]. Binocular testing was done first and depending on the patient’s attention and cooperation levels, monocular testing was attempted later. The test was performed in ambient room lighting.

#### 2.3.3. Baby vision screener (BaViS).

**Fig 1:**
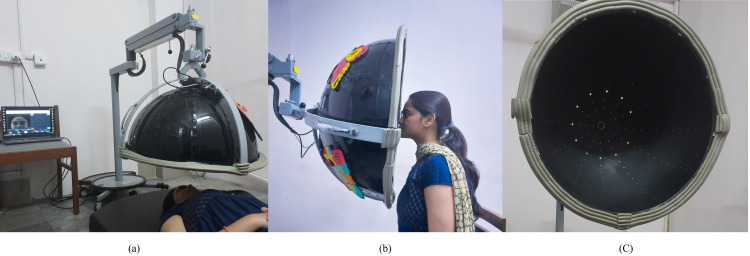
Visual field being quantified using BaViS in (a) supine position and (b) sitting position. (c) View inside the dome showing the LED arrays. The actual testing is done in a dark room.

The BaViS (earlier called as Pediatric Perimeter) used in this study is described elsewhere (https://youtu.be/Lk8jSvS3thE) [11,16]. Briefly, the device consists of a hemispherical dome (Fig 1, the individual pictured in the figure has provided written informed consent (as outlined in PLOS consent form) to publish their image alongside the manuscript.) with Light Emitting Diodes (LED) placed at different cardinal meridians (12 meridians, starting from 0° to 330°, placed at 30° intervals). These LEDs are controlled through a computer using custom-built software. Both static and kinetic targets can be presented in this device. Static targets consist of Quadrant and Meridian stimulus arrays. When LED arrays are presented in two adjacent meridians it is called Quadrant and when only one meridian is presented, it is called Meridian target. In both these targets, the LEDs are presented from an eccentricity of 30° to 90° from the center of the dome. The targets remain “on” till the patient looks at the target or for 5 seconds, after which the target is turned off. The Kinetic target consists of a single LED lighting up sequentially along the meridian from the periphery (90°) toward the center of the device. The speed of this stato-kinetic presentation was set for 6°/sec. The infrared camera mounted at the apex of the dome captures the patient’s face and presents it on a graphical user interface (GUI) for the examiner to observe and record the patient’s response in real-time. The patient’s eye and/or head movement towards the presented target is considered as a response. This response is recorded with a key press by the examiner. Through the GUI, the examiner can also present different targets. The kinetic target is used to quantify the visual field extent. The static targets (Quadrants and Meridians) are used to screen for gross visual field defects when the quantification of the visual field extent is not possible. In patients with shorter attention span, doing a Kinetic test can be quite challenging. Patients can be positioned either in a supine or in a seated position, and the dome can be flipped accordingly.

During examination, the examiner was masked to the outcomes of other perimetric tests, if it had been performed. For those patients on whom more than one test was performed, different examiners (authors MT, PNS, or a research optometrist) performed the test, to avoid any bias. Such masking is practiced for any patient tested on this device as a routine.

### 2.4. Data analysis

As this was an observational study to investigate the feasibility of testing with different perimetric tests, no formal statistical tests were performed. The outcome measure was the number of patients who were able to perform the tests. When possible, the outcome visual field isopters between devices were qualitatively compared.

## 3. Results

### 3.1. Clinical examination

The mean age ±  standard deviation of the patients was 18 ±  8.7 years (age range: 6 years to 38 years, 12 males and 5 females). The clinical findings of these 17 patients are given in Table 1. Visual acuity was measured for all the patients except one, (case 9, Table 1) for whom the family members wanted only the visual fields to be tested and no other tests. Of the 16 patients tested, 7 (44%) patients had reduced visual acuity (20/30 or worse) in each eye even after refractive correction. Ocular alignment was assessed for 15 patients. Strabismus (exodeviation) was observed in 53% of the tested patients and nystagmus in 20% of the patients.

**Table 1:** Clinical profile of the patients diagnosed with drug-resistant epilepsy.

Case	Age/Gender	MRI findings	Visual acuity	Ocular alignment	Other eye findings	Confrontation Examination	HFA	Tangent screen perimetry	BaViS
1	25/M	WNL	RE: 20/20 LE: 20/20	Ortho	WNL	Normal	30-2: advanced field defect, high FN and FL	NVFD	Not available
2	35/F	WNL	RE: 20/50 LE: 20/632	LXT	NA	Normal	30-2: advanced field defect, high FN and FL	NVFD	Not available
3	17/M	Right occipital infarct with gliotic changes	RE: 20/40 LE: 20/125	LXT	WNL	Normal	30-2: advanced field defect, high FN and FL	NVFD	Not available
4	23/M	Subcortical white matter changes in bilateral parieto-occipital area	RE: 20/25 LE: 20/20	IXT	Partial optic atrophy	Uncooperative	Unable to perform	NVFD	Not available
5	19/M	Bilateral parieto-occipital white matter hyperintensities	RE: 20/25 LE: 20/30	LXT	WNL	Uncooperative	Unable to perform	NVFD	Not available
6	16/M	Gliosis, volume loss in occipital and parietal lobe	RE: 20/25 LE: 20/30	IXT	Partial optic atrophy	Uncooperative	Unable to perform	RE- restricted inferonasal quadrant LE-NVFD	RE- restricted inferonasal quadrant LE-NVFD
7	16/M	Gliotic changes in right parietal, occipital, and left parietal lobe	RE: 20/30 LE: 20/40	Ortho	Partial optic atrophy	Left hemianopia	30-2: BE: left peripheral defect, 60-4 monocular: BE advanced field defect	BE: restricted left inferior quadrant	BE: restricted left inferior quadrant
8	9/F	WNL	RE: 20/20 LE: 20/20	Ortho	WNL	Uncooperative	Unable to perform	Unable to perform	NVFD
9	15/F	Left parieto-occipital gliosis	NA	NA	NA	Right hemianopia	Unable to perform	Not attempted	Right homonymous hemianopia
10	18/F	Left parieto-tempro-occipital gliosis. Diagnosed case of MELAS	RE: 20/25 LE: 20/25	End gaze nystagmus	NA	Uncooperative	Unable to perform	Not attempted	Right hemifield loss (right neglect noted)
11	13/M	Bilateral parieto-occipital gliosis	RE: 20/80 LE: 20/60	RXT, FMN	WNL	Uncooperative	Unable to perform	Not attempted	BE: restriction of inferior field
12	6/M	Hyperintense gliotic changes in the bilateral parieto-occipital area	RE: 20/50 LE: 20/50	XT	Optic atrophy	Uncooperative	Unable to perform	Not attempted	RE: constriction of nasal visual field LE: constriction of nasal and superior visual field
13	14/M	Sequelae of perinatal/neonatal hypoxic insult	RE: 20/20 LE:20/20	NA	Exophoria	Uncooperative	30-2: high FN and FL, 120-2: BE: relative peripheral and mid-peripheral defect	Not attempted	Binocularly NVFD
14	11/F	Right median occipital and parietal gliosis	RE: 20/30 LE: 20/30	Ortho	WNL	Normal	30-2: high FN, Esterman binocular suprathreshold test: relative left inferior field	Not attempted	Binocularly NVFD
15	38/M	Volume loss in the right hippocampus, hyperintensity in the right occipital lobe	RE: 20/20 LE: 20/20	Ortho	WNL	Normal	kinetic mode: RE, LE: constriction (1^st^ visit, 2^nd^ visit only RE – inferior constriction)	Not attempted	LE: NVFD (both visits), RE: some inferior field constriction (both visits)
16	23/M	Bilateral parieto-occipital gliosis	RE: 20/40 LE: 20/50	LXT, rotatory nystagmus	NA	Uncooperative	30-2: advanced field defect with high FN and FL	Not attempted	NVFD
17	8/M	Gliotic changes in bilateral occipital and right temporal lobes	Binocular: 20/40	NA	NA	Uncooperative	Unable to perform	Not attempted	Grossly NVFD

HFA, Humphrey Visual Field Analyzer; BaViS, Baby Vision Screener; RE, right eye; LE, left eye; BE, both eyes; XT, exotropia; IXT, intermittent exotropia; WNL, within normal limits; NA, not assessed; FN, false negative; FL, fixation loss; FMN, fusion-maldevelopment nystagmus; NVFD, no visual field defect; MELAS, Mitochondrial encephalomyopathy lactic acidosis and stroke like episodes.

### 3.2. Visual field examination

HFA was first attempted in all the patients. Among the seventeen patients, 47% (8/17) of the patients could be tested using HFA and the other patients were unable to perform this test. However, within those tested, reliable visual field measures could be obtained only in two patients. In the remaining patients, the reliability indices of false negatives and fixation losses were very poor (Table 1), indicating that the test results were unreliable. Throughout the test, the examiner was repeatedly encouraging and explaining the instructions to the patient and closely monitored the performance of the patient. Patients were also given multiple breaks during the test. On average the entire testing duration with the break took about 30-40 minutes with breaks.

Not all the patients were tested with the alternative visual field tests (tangent screen perimetry and BaViS). The BaViS device was unavailable for earlier patients, as it was under development at that time. In 3 patients both the alternative devices, along with HFA were tested. In tangent screen perimetry out of the eight patients, only one could not perform the test. With the BaViS, in 11 of the 12 patients, the visual field isopters could be plotted. For the remaining patient (case 17, Table 1) only a gross measure of the visual field assessment was possible with the BaViS. The gross measure essentially indicates the presence or absence of quadrantanopia or hemianopia.

**Fig 2:**
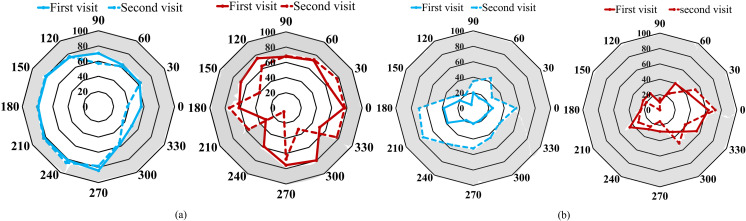
Visual field isopters of case 15 plotted using (a) BaViS and (b) HFA (kinetic mode), on two different days. The right eye is represented by red color and the left eye is represented by blue line. Note that the data from the original HFA plot is taken to generate these visual field isopters to appreciate the overlap.

**Fig 3:**
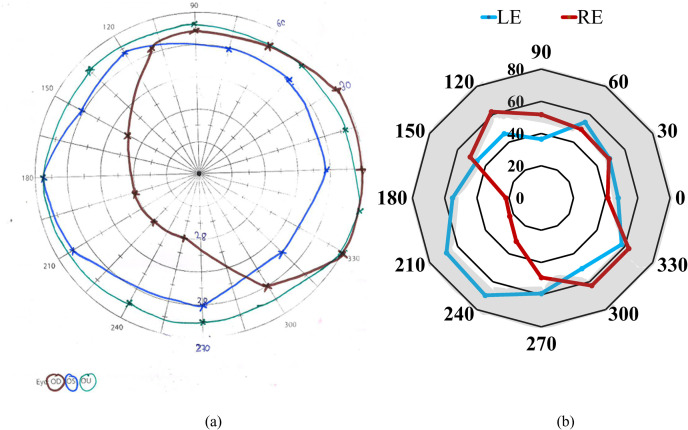
Visual field isopter of case 6 plotted using (a) Tangent screen and (b) BaViS. The visual field extent plotted with the tangent screen perimetry is 43° and for BaViS is 90°. The area inside the connected lines indicates the seeing visual field. Each eye’s visual field isopter is plotted in a different color. RE, OD =  Right eye (red line), LE, OS = Left eye (blue line), OU = Both eyes (plotted in the tangent screen perimetry in green line).

**Fig 4:**
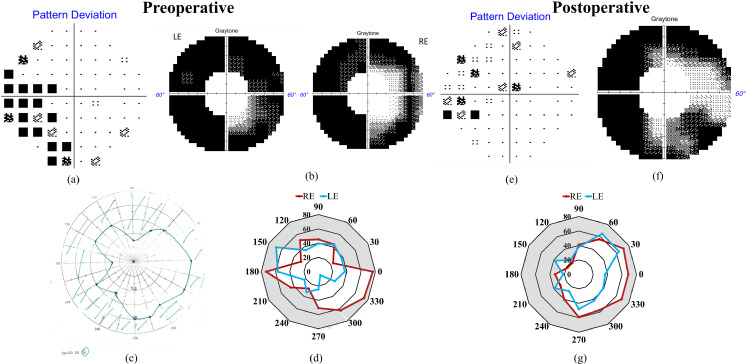
Preoperative (a–d) and post-operative (e–g) visual fields of case 7 tested with different perimertic techniques. (a) Preoperative pattern deviation of central 30° binocular visual field tested with 30-2 program of HFA. (b) Preoperative monocular visual fields tested with 60-4 program of HFA (c) Preoperative binocular visual field tested with tangent screen perimetry. (d) Preoperative monocular visual fields tested with BaViS (red color denotes right eye (RE) and blue denotes left eye (LE)). (e) Postoperative pattern deviation of central 30° binocular visual field with 30-2 program of HFA. (f) Postoperative binocular visual field tested with 60-4 program of HFA. (g) Postoperative monocular visual fields tested with BaViS.

One patient (case 15, Table 1) had visual field assessment with both BaViS and HFA, assessed 4 months apart. He had a constricted visual field (left eye) with the kinetic mode of HFA in the first visit, which could not be recorded in the follow-up visit. In both the visits, the visual fields in the right eye with HFA, and both eyes visual fields with the BaViS were reproducible (Fig 2). In the three patients for whom both BaViS and tangent screen perimetry were assessed, one patient could not perform the tangent screen perimetry. In the remaining two patients comparable visual field defects were observed with both these devices (Figs 3 and 4).

In one patient (case 7) visual field testing was possible with all the 3 devices. This patient also underwent surgery for his DR-PCE. Preoperatively, the binocular visual field was tested using HFA (30-2 program). Few relative depressed points were identified on the left side of the central 30° of visual field (Fig 4a). His monocular peripheral visual fields (beyond 30° from the point of fixation) were also tested using a 60-4 testing program of HFA, revealing advanced overall peripheral visual field defects in both eyes as shown in Fig 4b. Binocularly tangent screen perimetry showed constriction of the left inferior visual field (Fig 4c). Monocular BaViS testing showed constriction of the left inferior visual field in the right eye and overall inferior visual field constriction in the left eye. When the visual field isopters of both eyes obtained from the BaViS were overlapped, the pattern of visual field constriction was similar to that of tangent screen perimetry (Fig 4d). Visual field plots obtained from both tangent screen perimetry and BaViS did not match with the visual field obtained from the 60-4 testing program of HFA. The impaired visual field was larger in the 60-4 program as compared to the other techniques.

Postoperatively, the visual field was reevaluated binocularly using the 30-2 and 60-4 testing programs of HFA. Unlike the previous result, postoperatively no depressed visual field points were noted on the left side in the binocular central 30° of the visual field (Fig 4e). The binocular visual field tested with the 60-4 program, showed a left-side visual field defect (Fig 4f). When tested monocularly with BaViS, constriction of the left side visual field beyond 30° was observed in both the left and right eye suggestive of left homonymous hemianopia (Fig 4g).

## 4. Discussion

This study highlights that 88% (15 out of 17) of patients with DRE who were referred to our clinic were either unable to perform the visual field test or if tested had unreliable test results with the conventional device (HFA). With the clinical confrontation examination technique about 60% (10 out of 17) could not cooperate for the test (Table 1). Therefore, these patients required alternative techniques to assess their visual fields. This gap can be addressed by devices like tangent screen perimeter and the recently developed BaViS. Visual field quantification was possible in 91% (11/12) of these patients when tested with BaViS (Table 1). In one child diagnosed with attention deficit hyperactive disorder (case 17), the Kinetic test could not be performed. However, gross visual fields with static targets could still be tested to check for dense hemianopia or quadrantanopia. Thus, in all patients (100%) at least a gross visual field estimate is possible with BaViS. With tangent screen perimetry, 87% (7/8) were testable. The current testability of the BaViS suggests that this device is valuable for quantifying the visual field extent in those patients who cannot perform conventional testing.

The conventional automated static perimetry using HFA requires patient concentration, cooperation, stable head position on the chinrest, steady eye fixation for prolonged time-period in a dark room, and a button press to register the response. These prerequisites make it a cognitively challenging task for most patients, particularly children and older patients, even those without any cognitive impairment. They find this task difficult and tiring [17]. Unlike manual perimetry, automated static perimetry diminishes the role of the perimetrist in patient performance surveillance [18,19]. Multiple catch trials (false positive, false negative, and fixation loss) are introduced to measure the reliability of the test. High false positive indicates “trigger-happy” patients and the subsequent test result may show no field defect, whereas high rates of false negatives indicate “inattentive” patients, and the subsequent test result could show an advanced field defect [19]. In the current study, advanced visual field defects were observed in the majority of the patients (75%) tested with the 30-2 testing program because of the high false negative (Table 1). Beyond inattention, visual fatigue and diminished alertness in patients with epilepsy could have also resulted in this unreliable result, with high fixation losses [20]. To minimize this challenge, with assiduity, the perimetrist should closely monitor patients with epilepsy compared to cognitively healthy patients, reemphasize instructions throughout the test and provide multiple breaks to alleviate test-induced fatigue. As a result, when these criteria were followed in the current study for three patients tested with the 60-4, 120-2, and Esterman binocular test, the reliability indices improved. Such a solution of providing multiple breaks with constant monitoring will prolong the test duration and may not be practical, particularly in busy clinics. Patients may get tired due to the prolonged test duration. Also, the purpose of the “automation” is lost if it requires constant supervision by the perimetrist.

In such a scenario exploring alternative methods such as tangent screen perimetry and BaViS are worthwhile pursuits. Both these methods eliminate the need for a stable head position on a chin rest and the need to press a button after target appearances, thereby making the task less demanding. Also, the targets used in these tests are suprathreshold-only and are closer to real-world targets, unlike the tiny spot of light that is presented in the HFA. However, it must be noted that HFA can measure thresholds and so give the depth of the visual field loss. In contrast, with suprathreshold targets only absolute but not relative defects can be observed. This could explain the variability in different tests seen in case 7 (Fig 4). In automated perimeters, the test cannot be modified on the fly after it has begun, whereas in the alternative methods, the perimetrist can modify the order of target presentation during testing, based on the individual’s performance. In addition to assessing visual field extent, BaViS also quantifies reaction time to peripheral stimuli. Studies demonstrate that patients with glaucoma exhibit delayed reaction times corresponding to their visual field defects [21–25]. Eye movement perimetry is also shown to be helpful in children with intracranial lesions [26]. Therefore, alternative techniques looking at reaction time and eye movements shows promise for assessing functional integrity of the visual field.

Campimetry has been used to quantify the visual field in children with epilepsy [27]. In principle, tangent screen perimetry is similar to Campimetry. A verbal response is needed from the patient in these methods, to indicate target detection [14]. This could be a challenge for those patients who will be unable to give that response. The perimetrist can observe the patient’s eye movement toward the target as a surrogate measure for detection. But this again will be a challenge, in those patients who are unable to maintain steady fixation, and frequently search for the target which might induce false positive results. For those patients who are unable to sit for the duration of the test, using these devices will also be a challenge. Additionally, the visual field extent that can be measured with the tangent screen perimetry is limited. These challenges can be overcome to some extent with BaViS. Patients can be tested in both supine and seated position. In the cohort of our patients, 91% were tested in the supine position. Head stability is better in the supine position and the peripheral distractors are also cut off because of the dome of the BaViS device. The BaViS requires less time to quantify visual field extent. The entire test took 5–10 minutes in this study cohort. As patients’ reflexive eye movements toward the peripheral target are monitored and video recorded, the test video can also be reviewed if in doubt about the fixation loss. Unlike the tangent screen perimetry, the target speed of the BaViS and HFA is constant. This reduces the variation in target presentation speed between examiners, which is a limitation in the tangent screen perimetry. In our study, we found that all patients were able to perform the test with BaViS. BaViS appeared to have good repeatability, but this could only be shown in one patient (case 15). The better repeatability in this patient could be due to the ease of the test that encourages reflexive fixation. Further studies are needed to understand the repeatability of BaViS with a larger cohort. Other alternative techniques such as objective perimetry using pupil responses are also promising for patients with epilepsy [28] and the time taken to assess the visual fields are also rapid with this method [29].

Visual field defects were noted in 36% of the tested patients with tangent screen perimetry and BaViS. In cases where both tangent screen perimetry and BaViS were used to assess the visual field extent both the devices revealed comparable visual field defects. Since tangent screen perimetry is a well-established technique to quantify neurological visual field defects [30,31], obtaining similar results from both techniques is encouraging for BaViS. In one patient (case 7) a left homonymous hemianopia was documented with both HFA and BaViS. This hemianopic visual field defect in this patient also correlated with his presenting complaints. This finding indicates BaViS will be a valuable tool for identifying hemianopic visual field defects in patients with epilepsy. HFA showed a larger area of visual field defect when compared with the alternative perimetric techniques (BaViS and tangent screen perimetry) in one patient (case 7). This could have occurred because of the supra-threshold targets used in the alternative perimetric techniques. These targets are not sensitive to identify the relative depth of visual field defects. This is a limitation of these devices. It must be noted that BaViS and tangent screen perimetry can mostly detect neurological types of visual field defects and are limited in detecting central scotoma or enlarged blind spots, which can appear in individuals with raised intracranial pressure [32,33], or glaucoma [34]. These defects can only be detected using conventional automated perimetry, like HFA. However, as BaViS has software and hardware components, it can be modified to present light stimuli of lower or higher intensity and as static stimuli as well. The other possible reason for the discrepancy between HFA, and other techniques could also be the phenomenon of stato-kinetic dissociation. The patients might have perceived moving but not stable targets. Because of stato-kinetic dissociation, the margins of field loss detected by Humphrey (static Perimetry) can be larger as compared to the field loss detected by Kinetic perimetry [35]. Nonetheless, the primary objective of assessing visual field defects in patients with epilepsy is to ascertain whether the presence of these defects affects their ability to perform daily living tasks. To measure such a functional visual field, both tangent screen perimetry and BaViS could be useful.

This study also reports the ophthalmic clinical profile of patients with epilepsy. Visual fields were intact in 64% of the tested patients, which is similar to the prevalence reported in a studies done on adults and children with epilepsy [27,36,37]. Exodeviation was observed in 53% of the tested patients with DR-PCE. Exotropia has been reported in individuals with neurological damage [38]. Subnormal vision was observed in 43% of the tested patients. For a few patients (cases 6, 7, and 12) the subnormal vision is explained by the presence of the optic atrophy. For others, the reason for subnormal vision needs further evaluation.

This study had a few limitations. A small sample size should be considered when generalizing the results of this study. Additionally, these patients were referred from the neurology clinic, and hence there is a selection bias to this sample, and they are not representative of all DRE patients in general. However, since the scope of this study was to demonstrate alternative techniques to conventional perimetry, the selection bias is not a major limitation. Not all patients were tested in all the devices, to make comparisons between the devices. The retrospective nature of this study was also a reason for this, along with the practical difficulty that these patients may not endure many tests in a single visit. However, the study does show the feasibility of using alternative perimetric techniques to estimate the visual fields. While there were no control subjects in this group, an earlier study [11] showed comparable visual fields with HFA and earlier version of BaViS on patients without epilepsy.

## 5. Conclusion

In conclusion, the BaViS and tangent screen perimeters can be used to assess the visual fields in patients with epilepsy, who are unable to do the conventional perimetry. BaViS allows testing a wide angle of the visual field (>50°) that is limited in other perimetry techniques such as HFA, Campimetry, and tangent screen perimetry. Detection of visual field defects preoperatively will be helpful for the neurosurgeon to plan the surgery and also to counsel the patients and caregivers regarding the visual prognosis. Postoperative detection of new visual field deficits will also help the clinician counsel the patient and plan for rehabilitation if needed. Further studies will be required with more patients to determine the repeatability of these alternative perimeters.

## References

[1] Kwan P, Schachter SC, Brodie MJ. Drug-resistant epilepsy. N Engl J Med. 2011;365(10):919–26. doi: 10.1056/NEJMra1004418 21899452

[2] Löscher W, Schmidt D. Modern antiepileptic drug development has failed to deliver: ways out of the current dilemma. Epilepsia. 2011;52(4):657–78. doi: 10.1111/j.1528-1167.2011.03024.x 21426333

[3] Golyala A, Kwan P. Drug development for refractory epilepsy: The past 25 years and beyond. Seizure. 2017;44:147–56. doi: 10.1016/j.seizure.2016.11.022 28017578

[4] Luders H, Engel J, Munari C. Noninvasive preoperative evaluation: general principles. In: Engel J Jr, editor. Surgical treatment of the epilepsies. 2nd ed. New York: Raven Press; 1993.

[5] Siegel AM. Presurgical evaluation and surgical treatment of medically refractory epilepsy. Neurosurg Rev. 2004;27(1):1–18; discussion 19-21. doi: 10.1007/s10143-003-0305-6 14586764

[6] Sierra-Marcos A, Fournier-Del Castillo MC, Álvarez-Linera J, Budke M, García-Fernández M, Pérez-Jiménez MA. Functional surgery in pediatric drug-resistant posterior cortex epilepsy: electro-clinical findings, cognitive and seizure outcome. Seizure. 2017;52:46–52. doi: 10.1016/j.seizure.2017.09.013 28963933

[7] Blume WT, Whiting SE, Girvin JP. Epilepsy surgery in the posterior cortex. Ann Neurol. 1991;29(6):638–45. doi: 10.1002/ana.410290611 1892366

[8] Dalmagro CL, Bianchin MM, Velasco TR, Alexandre V Jr, Walz R, Terra-Bustamante VC, et al. Clinical features of patients with posterior cortex epilepsies and predictors of surgical outcome. Epilepsia. 2005;46(9):1442–9. doi: 10.1111/j.1528-1167.2005.70904.x 16146440

[9] Kun Lee S, Young Lee S, Kim D-W, Soo Lee D, Chung C-K. Occipital lobe epilepsy: clinical characteristics, surgical outcome, and role of diagnostic modalities. Epilepsia. 2005;46(5):688–95. doi: 10.1111/j.1528-1167.2005.56604.x 15857434

[10] Grewal SS, Tatum WO, Brazis PW, Shih JJ, Wharen RE. Preoperative visual field deficits in temporal lobe epilepsy. Epilepsy Behav Case Rep. 2017;7:37–9. doi: 10.1016/j.ebcr.2016.12.005 28348961 PMC5357740

[11] Satgunam P, Datta S, Chillakala K, Bobbili KR, Joshi D. Pediatric perimeter-A novel device to measure visual fields in infants and patients with special needs. Transl Vis Sci Technol. 2017;6(4):3. doi: 10.1167/tvst.6.4.3 28685105 PMC5497602

[12] Satgunam P, Thakur M, Saha R, Nagarajan K, Singh H, Murugvel M. Baby Vision Screener (BaViS): A device to quantify infant’s fixation to light. In: American Academy of Optometry Conference. New Oreleans; 2023.

[13] Thakur M, Chattannavar G, Satgunam P. Hemianopic visual field loss in a young child tested with pediatric perimeter: a case report. Asian J Physics. 2023;32:167–72.

[14] McLean AJ. practical perimetry: construction and operation of the tangent screen. Can Med Assoc J. 1937;36(6):578–83. 20320634 PMC1562237

[15] Esterman B. Functional scoring of the binocular field. Ophthalmology. 1982;89(11):1226–34. doi: 10.1016/s0161-6420(82)34647-3 7155532

[16] Thakur M, Chattannavar G, Saha R, Singh H, Kekunnaya R, Satgunam P. Unraveling congenital ptosis with the aid of the pediatric perimeter device. Indian J Ophthalmol. 2023;71(3):1058. doi: 10.4103/ijo.IJO_2915_22 36872759 PMC10229992

[17] Gardiner SK, Demirel S. Assessment of patient opinions of different clinical tests used in the management of glaucoma. Ophthalmology. 2008;115(12):2127–31. doi: 10.1016/j.ophtha.2008.08.013 19041473 PMC3704561

[18] Heijl A, Krakau CE. An automatic static perimeter, design and pilot study. Acta Ophthalmol (Copenh). 1975;53(3):293–310. doi: 10.1111/j.1755-3768.1975.tb01161.x 1174394

[19] Frankhauser F, Spahr J, Bebie H. Some aspects of the automation of perimetry. Surv Ophthalmol. 1977;22(2):131–41. doi: 10.1016/0039-6257(77)90094-7 335547

[20] Katz J, Sommer A. Reliability indexes of automated perimetric tests. Arch Ophthalmol. 1988;106(9):1252–4. doi: 10.1001/archopht.1988.01060140412043 3046588

[21] Mazumdar D, Pel JJM, Panday M, Asokan R, Vijaya L, Shantha B, et al. Comparison of saccadic reaction time between normal and glaucoma using an eye movement perimeter. Indian J Ophthalmol. 2014;62(1):55–9. doi: 10.4103/0301-4738.126182 24492502 PMC3955071

[22] Kadavath Meethal NS, Mazumdar D, Asokan R, Panday M, van der Steen J, Vermeer KA, et al. Development of a test grid using eye movement perimetry for screening glaucomatous visual field defects. Graefes Arch Clin Exp Ophthalmol. 2018;256(2):371–9. doi: 10.1007/s00417-017-3872-x 29282563 PMC5790865

[23] Trope GE, Eizenman M, Coyle E. Eye movement perimetry in glaucoma. Can J Ophthalmol. 1989;24(5):197–9. 2766083

[24] Kanjee R, Yücel YH, Steinbach MJ, González EG, Gupta N. Delayed saccadic eye movements in glaucoma. Eye Brain. 2012;4:63–8. doi: 10.2147/EB.S38467 28539782 PMC5436190

[25] Lamirel C, Milea D, Cochereau I, Duong M-H, Lorenceau J. Impaired saccadic eye movement in primary open-angle glaucoma. J Glaucoma. 2014;23(1):23–32. doi: 10.1097/IJG.0b013e31825c10dc 22706338

[26] Meethal NSK, Robben J, Mazumdar D, Loudon S, Naus N, Polling JR, et al. Detection of visual field defects using Eye Movement Pediatric Perimetry in children with intracranial lesions: feasibility and applicability. Heliyon. 2022;8(11):e11746. doi: 10.1016/j.heliyon.2022.e11746 36419654 PMC9676553

[27] Neumayr L, Pieper T, Kudernatsch M, Trauzettel-Klosinski S, Staudt M. Uncovering homonymous visual field defects in candidates for pediatric epilepsy surgery. Eur J Paediatr Neurol. 2020;25:165–71. doi: 10.1016/j.ejpn.2019.11.003 31784289

[28] Ali EN, Lueck CJ, Carle CF, Martin KL, Borbelj A, Maddess T. Response characteristics of objective perimetry in persons living with epilepsy. J Neurol Sci. 2022;436:120237. doi: 10.1016/j.jns.2022.120237 35358854

[29] Maddess T, van Kleef JP, Rohan EMF, Carle CF, Baird-Gunning J, Rai BB, et al. Rapid, non-contact multifocal visual assessment in multiple sclerosis. Neurol Sci. 2023;44(1):273–9. doi: 10.1007/s10072-022-06387-z 36098887 PMC9816274

[30] Wong AM, Sharpe JA. A comparison of tangent screen, goldmann, and humphrey perimetry in the detection and localization of occipital lesions. Ophthalmology. 2000;107(3):527–44. doi: 10.1016/s0161-6420(99)00092-5 10711892

[31] Keltner JL, Johnson CA. Automated and manual perimetry-a six-year overview. Special emphasis on neuro-ophthalmic problems. Ophthalmology. 1984;91(1):68–85. doi: 10.1016/s0161-6420(84)34328-7 6709321

[32] Kedar S, Ghate D, Corbett JJ. Visual fields in neuro-ophthalmology. Indian J Ophthalmol. 2011;59(2):103–9. doi: 10.4103/0301-4738.77013 21350279 PMC3116538

[33] Wall M, George D. Idiopathic intracranial hypertension. A prospective study of 50 patients. Brain. 1991;114(Pt 1A):155–80. 1998880

[34] Chakravarti T, Moghimi S, De Moraes CG, Weinreb RN. Central-most visual field defects in early glaucoma. J Glaucoma. 2021;30(3):e68–75. doi: 10.1097/IJG.0000000000001747 33273288

[35] Safran AB, Glaser JS. Statokinetic dissociation in lesions of the anterior visual pathways. A reappraisal of the Riddoch phenomenon. Arch Ophthalmol. 1980;98(2):291–5. doi: 10.1001/archopht.1980.01020030287009 7352878

[36] Liava A, Mai R, Cardinale F, Tassi L, Casaceli G, Gozzo F, et al. Epilepsy surgery in the posterior part of the brain. Epilepsy Behav. 2016;64(Pt A):273–82. doi: 10.1016/j.yebeh.2016.09.025 27788449

[37] Yu T, Wang Y, Zhang G, Cai L, Du W, Li Y. Posterior cortex epilepsy: diagnostic considerations and surgical outcome. Seizure. 2009;18(4):288–92. doi: 10.1016/j.seizure.2008.11.008 19136282

[38] Good WV, Jan JE, DeSa L, Barkovich AJ, Groenveld M, Hoyt CS. Cortical visual impairment in children. Surv Ophthalmol. 1994;38(4):351–64. doi: 10.1016/0039-6257(94)90073-6 8160108

